# Effects of Intestinal Microbiota on Individual Growth and Development of *Pontastacus leptodactylus*

**DOI:** 10.3390/microorganisms14081739

**Published:** 2026-08-07

**Authors:** Mengjie Pi, Bin Li, Haorui Wang, Qi Zhao, Xiaosen Li, Zhenhua Du, Yizhe Wang, Na Li, Ruiyang Xia, Yang Zhang, Qingyong Guo

**Affiliations:** 1 Parasitology Laboratory, College of Veterinary Medicine, Xinjiang Agricultural University, Urumqi 830052, China; 2 Xinjiang Key Laboratory of New Drug Research and Development for Herbivorous Animals (XJ-LNDRDHA), Urumqi 830052, China; 3 Xinjiang Regional Key Laboratory of Clinical Veterinary Medicine Research (XJRKLCVMR), Urumqi 830052, China; 4 College of Animal Science and Technology, Xinjiang Agricultural Vocational and Technical University, Changji 831100, China

**Keywords:** *Pontastacus leptodactylus*, growth performance, intestinal microbiota, 16S rRNA sequencing

## Abstract

*Pontastacus leptodactylus* is an aquatic resource endemic to Xinjiang, and its growth performance is closely related to the composition and structure of its intestinal microbiota. This study used male *P. leptodactylus*, divided into a high-growth-performance (HGP) group and a low-growth-performance (LGP) group based on growth rates, to analyze differences in intestinal microbiota using microbial sequencing technology. Results showed that the number of ASVs unique to the HGP group (7840, accounting for 65.97%) was substantially higher than that in the LGP group (3383, 28.46%). ALDEx2 analysis identified six ASVs significantly enriched in the LGP group, assigned to *Methylobacterium*, *Devosia*, order Rhizobiales, *Renibacterium*, *Stenotrophomonas*, and class Mollicutes, which were near-absent in the HGP group. At the phylum level, TM7 was differentially abundant with higher relative abundance in HGP. At the genus level, ten genera showed significant differences: Pseudomonas, *Hydrogenophaga*, *Mesorhizobium*, *Reyranella*, *Mycobacterium*, *Flavobacterium*, *Ralstonia*, *Bradyrhizobium*, and *Aeromicrobium* were enriched in HGP, whereas the opportunistic pathogen *Plesiomonas* was depleted. Alpha diversity analysis revealed significantly higher Chao1, observed species, Shannon, and Faith’s pd indices in the HGP group (*p* < 0.05). Beta diversity analysis confirmed significant separation in microbial community structure between the two groups (adonis *p* = 0.018). KEGG pathway analysis showed upward trends in retinol metabolism, shigellosis, geraniol degradation, and xenobiotics metabolism pathways in HGP (*p* < 0.10), and a significant upregulation of coumarin biosynthesis (*p* < 0.01). COG analysis revealed eight downregulated functional genes in HGP (*p* < 0.01), suggesting more complex host–microbiota interactions. This study identifies distinct intestinal microbial signatures associated with growth performance in *P. leptodactylus*, providing candidate targets for probiotic development and aquaculture management, while causal verification remains the critical next step.

## 1. Introduction

*Pontastacus leptodactylus*, also known as the Irtysh River crayfish, is an endemic cold-water crayfish species whose natural distribution in the Xinjiang section of the Irtysh River basin was first documented in August 2023, and it plays a significant role in overwintering aquaculture [1]. Characterized by its large size, high meat yield, and delicious flavor, *P. leptodactylus* fills a gap in the known natural distribution of native crayfish in Xinjiang and holds considerable ecological, economic, and research value.

However, these changes have yet to be systematically analyzed in the specific species of *P. leptodactylus*. In artificial aquaculture, enhancing growth performance is key to improving farming efficiency. Beyond genetic, environmental, and nutritional factors, animal growth performance has recently been shown to be closely associated with the host intestinal microbiota [2]. The intestinal microbiota contributes to the production of short-chain fatty acids and vitamins, resistance to pathogen colonization, and the development and maintenance of immunity [3,4], and manipulating its composition can modulate host growth performance. For example, feeding *Litopenaeus vannamei* fermented feed containing *Bacillus subtilis* and *Lactobacillus acidophilus* increased survival and body-length growth rates while lowering the feed conversion ratio [5], and dietary supplementation with effective microorganisms (EM; lactic acid bacteria, photosynthetic bacteria, actinomycetes, and yeast) significantly improved weight gain rate, specific growth rate, and digestive enzyme activities in fish [6,7]. In other crustaceans, studies of *L. vannamei* [8], *Macrobrachium nipponense* [9], and *Procambarus clarkii* [10] have likewise associated intestinal microbiota structure with host growth, immunity, and metabolism, and further work indicates that the intestinal microbiota varies with feed ingredients, farming systems, and developmental stage [11,12,13,14,15,16,17].

Despite this progress, the intestinal microbiota of *P. leptodactylus* has not been systematically characterized, and comparisons among individuals differing in growth rate remain scarce. As a cold-water species endemic to the Irtysh River basin in Xinjiang, its distinctive ecological and physiological context may shape a particular intestinal microbiota, yet how this microbiota relates to growth is largely unexplored. Therefore, using high-throughput sequencing, this study investigates potential differences in the structural composition and functional characteristics of the intestinal microbiota between male *P. leptodactylus* individuals with high and low growth rates, aiming to provide a basis for understanding the association between growth and the intestinal microbiota in this species.

## 2. Materials and Methods

### 2.1. Experimental Animals and Rearing Conditions

This experiment was conducted at the breeding base for *P. leptodactylus* of the 187th Regiment in Altay, Xinjiang. The experimental animals were sourced from the same batch of outdoor pond-cultured populations. A total of 75 male *P. leptodactylus* were selected, with body weights ranging from 40~50 g. This study only selected male individuals to rule out the interference of sex-related physiological and microbiome variations on the growth–microbiome association analysis; at the same time, male *P. leptodactylus* show more noticeable differences in growth rate during farming and have higher market value, making them the main focus of breeding. All experimental individuals had intact appendages and chelae, no surface injuries, and normal responses to external stimuli. The water used for rearing the animals was sourced from the Irtysh River, consistent with the original farming water of the experimental population. During the rearing period, water quality conditions were stable, with the following specific parameters: water temperature 18~24 °C, pH ≥ 8.0, dissolved oxygen ≥ 7.5 mg/L, nitrite 0 mg/L, phosphate 0.058~0.071 mg/L, total hardness 117.205~140.448 mg/L, hydrogen sulfide 0 mg/L, residual chlorine < 0.08 mg/L, with the rearing environment under full natural light throughout the day.

The 75 experimental individuals were randomly assigned into 25 circular nylon hanging cages (Xiangxin Brand, China) in the pond (diameter 59 cm, height 62 cm), with 3 *P. leptodactylus* per cage. To reduce competition among individuals, individuals of similar body weight were placed in the same cage, and all cages were positioned at the normal water level for *P.* *leptodactylus* habitation. Prior to the experiment, exclusion criteria were established: individuals showing limb damage or abnormal vitality at the time of release were not included in subsequent statistics. During the experiment, due to environmental and weather influences, some individuals were lost, injured, escaped, or died, and ultimately 71 viable individuals (24 cages) were retained. After removing incomplete experimental cages, a total of 23 complete cages and 69 qualified individuals were obtained for subsequent phenotypic measurements and sequencing sample selection.

This study employed an extreme-phenotype sampling strategy for sample selection. Based on individual body weight increase (final body weight − initial body weight), the six individuals with the highest weight gain among the 69 qualified individuals were selected as the high-growth-performance (HGP) group, and the six individuals with the lowest weight gain were selected as the low-growth-performance (LGP) group. To avoid cage position effects and mitigate biases arising from non-independence of data from the same cage, 10 individuals from 10 independent hanging cages (HGP group A1–A5, LGP group B1–B5) were ultimately selected as sequencing samples.

Before the formal feeding experiment, all *P. leptodactylus* were acclimated in the experimental cages for 7 days. After acclimation, routine weighing and scheduled feeding were performed. The experimental feed consisted of frozen fish and a specially formulated *P. leptodactylus* feed developed by the base, mixed at a ratio of 3:2. The nutritional guaranteed values of the feed were as follows: crude protein ≥ 38%, crude fat ≥ 8.5%, crude fiber ≤ 8.0%, calcium ≥ 1.8%, total phosphorus ≥ 0.2%, magnesium ≥ 1.0%, lysine ≥ 2.5%, methionine ≥ 0.8%, crude ash ≤ 9.5%. The daily feeding amount was 3–5% of the total *P. leptodactylus* body mass in each cage, with feedings at 09:00 and 21:00 daily. Remaining feed was promptly removed after each feeding. During the rearing period, the molting status and health condition of the *P. leptodactylus* were observed and recorded daily, and feeding amounts were dynamically adjusted based on weather conditions, with normal feeding on sunny days and appropriately reduced amounts on rainy days. Before sample collection, *P. leptodactylus* were fasted for 24 h, and leftovers in the cages were thoroughly removed to ensure the intestinal contents of experimental individuals were emptied.

### 2.2. Collection of Intestinal Contents

On the day the experiment ended, the *P. leptodactylus* were fasted for 24 h. Afterwards, the experimental *P. leptodactylus* were taken out and transported to the lab under dry refrigeration at 4 °C for about 8 h. This low-temperature dry transport method is based on previous research on crustaceans [18], which can effectively suppress *P. leptodactylus* metabolism, reduce transport stress and individual suffering, and avoid secondary contamination of gut samples by microorganisms in the water. When the samples arrived at the lab, the *P. leptodactylus* were sedated on ice for 10 min. Euthanasia was performed painlessly by quickly crushing the ganglia under the cephalothorax with a sterile scalpel (Suqian Wenchuang Medical Devices Co., Ltd., Suqian, China), and complete death was confirmed by the absence of reflex movements when touching the eyestalks and appendages. After euthanasia, the surface of the *P. leptodactylus* was disinfected with 75% ethanol (Hais Hainuo Lexiang Medical Technology (Qingdao) Co., Ltd., Qingdao, China), and sterile dissection was conducted on an ice surface in a clean bench to isolate the complete gut tissue. Pre-cooled PBS solution was drawn with a 1 mL syringe to wash the gut, and 1 mL of the gut content eluent was collected into a 1.8 mL cryotube, with 500 μL of the eluent transferred into a new cryotube. Once all samples were processed, they were immediately stored at −80 °C for subsequent microbial DNA extraction and high-throughput sequencing analysis. All animal procedures in this experiment were approved by the Animal Ethics Committee of Xinjiang Agricultural University (Approval No.: 2024057). Throughout the experiment, low-temperature sedation and rapid central nervous system destruction were used for euthanasia, combined with multiple criteria for confirming death, fully complying with humane endpoints for experimental crustaceans.

### 2.3. Calculation Formulas for Growth Performance Indicator

Male *P. leptodactylus* were divided into the HGP group and LGP group based on the difference in body weight (final body weight − initial body weight). An independent-samples *t*-test was performed on the body weight differences in samples in the HGP and LGP groups using GraphPad Prism 10.1.2 (GraphPad Software, San Diego, CA, USA). Initial body weight (W0), final body weight (Wt), initial body length (L0), and final body length (Lt) were measured, and body length growth rate (BLGR, %), weight gain rate (WGR, %), specific growth rate (SGR, %/d), and survival rate (SR, %) were calculated [19].

(1) Body length growth rate (BLGR, %) = (Lt − L0)/L0 × 100

(2) Weight gain rate (WGR, %) = (Wt − W0)/W0 × 100

(3) Specific growth rate (SGR, %/d) = (lnWt − lnW0)/t × 100

(4) Survival rate (SR, %) = Xt/X0 × 100

### 2.4. 16S rRNA Sequencing

DNA Extraction: Total DNA from gut contents was extracted by Shanghai GenesBiotech Co., Ltd. (Shanghai, China) using the OMEGA Soil DNA Kit (D5635-02, Omega Bio-Tek, Norcross, GA, USA). Samples were added according to the kit’s recommended amounts and mechanically lysed using a 60 Hz grinder (Tissuelyser-48, Shanghai Jingxin, Shanghai, China). Finally, DNA was eluted with 100 μL Elution Buffer. The extracted DNA was quantified with a Nanodrop (NC2000, Thermo Scientific, Waltham, MA, USA) and checked for integrity using 1.2% agarose gel electrophoresis. For 12 samples, DNA concentrations ranged from 2.61 to 6.92 ng/μL, A260/280 ratios were 1.618–1.971, and no significant degradation was observed, meeting the library preparation requirements. Contamination Controls: No extraction blanks or negative controls at the sequencing level were set during DNA extraction quality control. To assess the risk of reagent/operation contamination, we used multiple pieces of evidence to argue that the detected genera are true gut signals: (1) all sample DNA concentrations were significantly higher than typical reagent contamination background (>2.5 ng/μL), and A260/280 ratios met purity standards; (2) sequencing company confirmed Qubit rechecks passed before library construction, 16S sequencing reads were sufficient (>40,000 per sample) and passed QC; (3) the discriminative genera in this study showed significant differences between the HGP and LGP groups, whereas uniform reagent contamination would appear equally across all samples; (4) representative sample gel electrophoresis bands were clear and single, without smearing or degradation (Supplementary Figure S2).

Shanghai Personal Biotechnology Co., Ltd. (Shanghai, China) was entrusted to extract DNA sequences of the 16S_V3V4 region from the intestinal microbiota of *P. leptodactylus* for amplification, with sequence lengths concentrated around 430 bp. The specific primer information included forward primer 338 F (5′-ACTCCTACGGGAGGCAGCA-3′) and reverse primer 806 R (5′-GGACTACHVGGGTWTCTAAT-3′), with a sample-specific 7 bp barcode incorporated into the primers for multiplex sequencing.

The PCR components contained 5 μL of buffer (5×), 0.25 μL of Fast pfu DNA Polymerase (5U/μL, TransGen Biotech, Beijing, China), 2 μL (2.5 mM) of dNTPs, 1 μL (10 μM) of each Forward and Reverse primer, 1 μL of DNA Template, and 14.75 μL of ddH_2_O. Thermal cycling consisted of initial denaturation at 98 °C for 5 min, followed by 26 cycles consisting of denaturation at 98 °C for 30 s, annealing at 52 °C for 30 s, and extension at 72 °C for 45 s, with a final extension of 5 min at 72 °C. PCR amplicons were purified with Vazyme VAHTSTM DNA Clean Beads (Vazyme, Nanjing, China) and quantified using the Quant-iT PicoGreen dsDNA Assay Kit (Invitrogen, Carlsbad, CA, USA). After the individual quantification step, amplicons were pooled in equal amounts, and pair-end 2 × 250 bp sequencing was performed using the Illumina NovaSeq platform with NovaSeq 6000 SP Reagent Kit (500 cycles) at Shanghai Personal Biotechnology Co., Ltd. (Shanghai, China).

### 2.5. Bioinformatics Analysis

Microbiome bioinformatics analysis was performed using QIIME2 (2019.4) with slight modifications according to the official tutorials. Raw paired-end sequences were demultiplexed with the demux plugin, and primers were trimmed using qiime cutadapt trim-paired. Each library was then independently processed with qiime dada2 (V1.1) denoise-paired for quality filtering, denoising, paired-end merging, and chimera removal. Truncation parameters (--p-trunc-len-f/r) were determined based on quality plots of each library (known values: run3_26P2288-1: f = 222/r = 227; run2_26P2292-2: f = 222/r = 230; values for the remaining libraries cannot be traced due to the 2024 project data being cleared). The maximum expected error rate was fixed at maxEE = 4.0, with other parameters kept as DADA2 defaults. After merging ASV feature sequences and ASV tables from all libraries, singleton ASVs (total count of 1 across all samples) were removed. The ASV table was rarefied to 95% of the minimum sample sequence count (51,931 sequences per sample) to normalize sequencing depth; rarefaction curves confirmed sufficient coverage for all samples.

Taxonomic classification was performed using the classify-sklearn algorithm with a Greengenes (Release 13.8) pre-trained Naive Bayes classifier at the default confidence threshold (0.7). A phylogenetic tree was constructed via the qiime phylogeny align-to-tree-mafft-fasttree workflow (MAFFT alignment, FastTree tree building).

Alpha diversity indices (Chao1, observed species, Shannon, Simpson, Faith’s PD, and Pielou’s evenness) were calculated from the rarefied ASV table. Beta diversity was assessed using Bray–Curtis distances and visualized with principal coordinate analysis (PCoA) and non-metric multidimensional scaling (NMDS). Community structure differences between groups were tested by PERMANOVA (adonis, distance~group, 999 permutations), and homogeneity of multivariate dispersion was examined with betadisper (permutest, 999 permutations).

Differential abundance analysis was performed using ALDEx2 with centered log-ratio transformation, the all-feature denominator, and 128 Monte Carlo instances. ASVs with Benjamini–Hochberg-adjusted *p* < 0.05 (wi.eBH < 0.05) were considered significantly differential.

Microbial functional potential was predicted using PICRUSt2 [19] (Nat Biotechnol 38:685–688; https://github.com/picrust/picrust2/wiki/, accessed on 31 October 2024) based on ASV feature sequences and their abundance tables, with functional pathway annotation against the MetaCyc (https://metacyc.org/ (accessed on 31 October 2024) and KEGG (https://www.kegg.jp/, accessed on 31 October 2024) databases. PICRUSt2 [20] infers functional gene content by phylogenetically placing each ASV against a reference genome database; sequences with a Nearest Sequenced Taxon Index (NSTI) > 2 are filtered by default to ensure prediction reliability. Differential pathways between groups were screened using the Mann–Whitney U test with BH correction (q < 0.05). It should be noted that the above functional analysis reflects genomic potential inferred from 16S rRNA gene sequences, rather than actual metabolic activity, mRNA expression, or enzyme activity. All functional-level interpretations are therefore presented as hypotheses to be verified.

### 2.6. Statistical Analysis

Data are expressed as mean ± standard deviation (Mean ± SD). Independent-samples *t*-test and non-parametric Mann–Whitney test were performed using GraphPad Prism 10.1.2 to analyze the significance of differences between the HGP and LGP groups. Normality of genus-level data was evaluated by the Shapiro–Wilk test (n = 5; data were considered non-normally distributed when *p* < 0.05), and homogeneity of variances was verified using Levene’s test. Genera meeting both normality and homoscedasticity assumptions were compared with independent-samples *t*-test; otherwise, the non-parametric Mann–Whitney U test was performed separately for each genus. Differential abundance analysis was implemented in ALDEx2, and significance was set at wi.eBH < 0.05 following Benjamini–Hochberg (BH) correction. The Benjamini–Hochberg (BH) procedure was used to control the false discovery rate (FDR) for all multiple comparisons, including genus-level taxa, predicted metabolic pathways, and COG functional categories, and adjusted q-values were presented. Differences with a *p*-value (or q-value) < 0.05 were regarded as statistically significant. *p* < 0.05 was considered statistically significant, and *p* > 0.05 indicated no significant difference.

## 3. Results

### 3.1. Determination of Growth Performance

All growth performance data are presented as mean ± standard deviation (mean ± SD). In this study, individual male *P. leptodactylus* were grouped into extreme phenotypes based on differences in body weight (final body weight − initial body weight), defined as the high-growth-performance (HGP) group and low-growth-performance (LGP) group, respectively. Since the two groups were selected based on the gradient of body weight differences, conducting inferential statistical tests for body weight difference, weight gain rate (WGR), or specific growth rate (SGR) creates a logical circularity. Therefore, these three indicators are presented only descriptively, without intergroup comparison tests or *p*-values. Statistical plotting of growth indicators was performed using GraphPad Prism 10.1.2.

The initial body weight, final body weight, and body weight difference in male *P. leptodactylus* samples in the HGP and LGP groups are presented in Table 1. Initial body length, final body length, and body length difference are shown in Table 2. Body length growth rate (BLGR, %), weight gain rate (WGR, %), and specific growth rate (SGR, %/d) are summarized in Table 3.

### 3.2. Composition and Structure of Intestinal Microbiota

#### 3.2.1. Venn Analysis of Intestinal Microbiota

**Figure 1:**
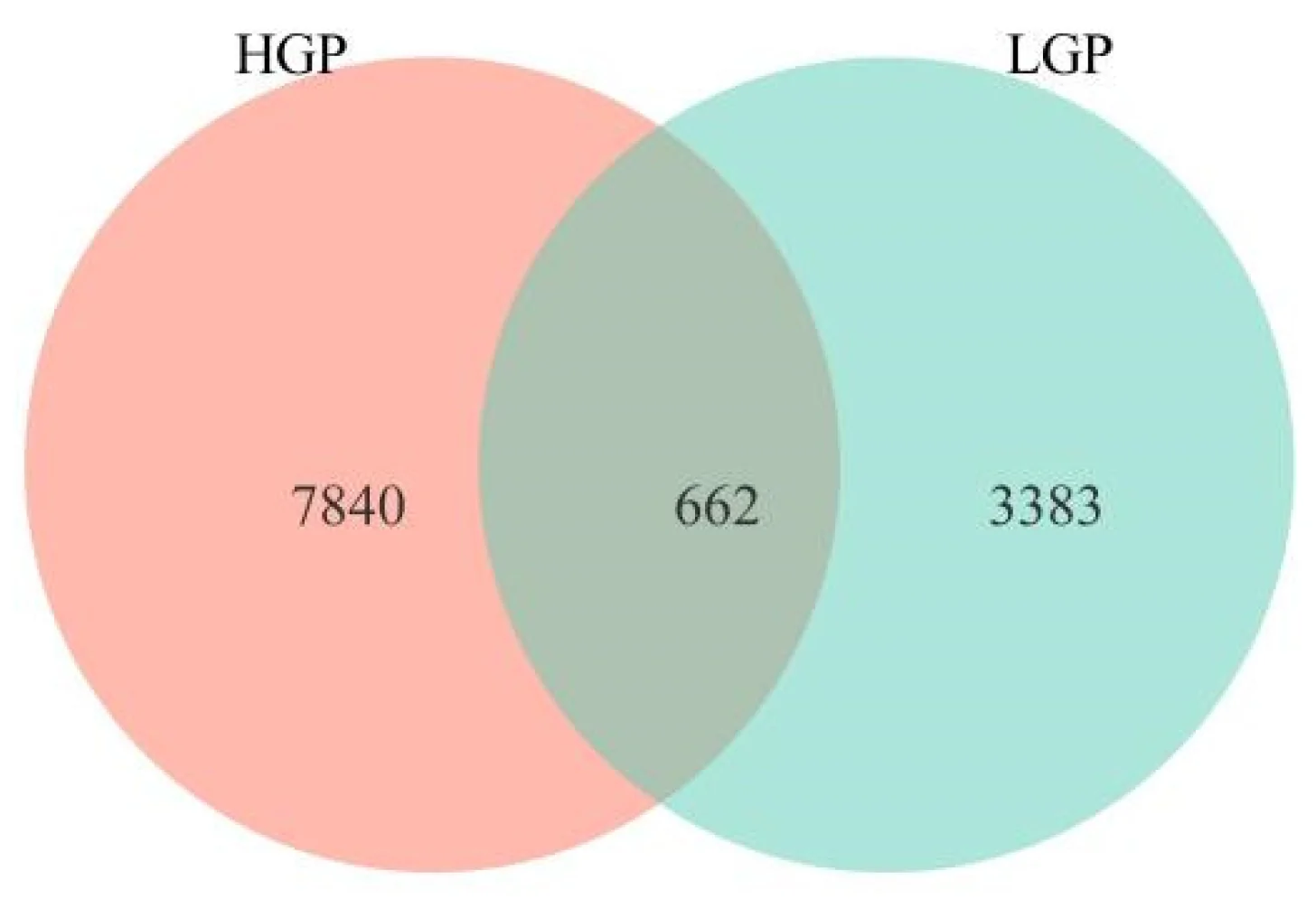
Venn analysis of intestinal microbiota ASVs in HGP and LGP groups. A total of 7840 ASVs were unique to the HGP group, 3383 ASVs were unique to the LGP group, and 662 ASVs were shared by both groups.

Venn analysis of the intestinal microbiota between the two groups (n = 5 each) revealed that the HGP group contained 7840 unique ASVs (65.97%), while the LGP group had 3383 unique ASVs (28.46%). The two groups shared 662 ASVs (5.57%) (Figure 1).

The HGP group exhibited remarkably high microbial specificity, with 7840 unique ASVs—substantially exceeding the 3383 in the LGP group and constituting over 60% of the total. This indicates the presence of numerous unique microbial taxa within the intestines of high-growth-performance *P. leptodactylus*.

#### 3.2.2. Associated Bacterial Identification

**Figure 2:**
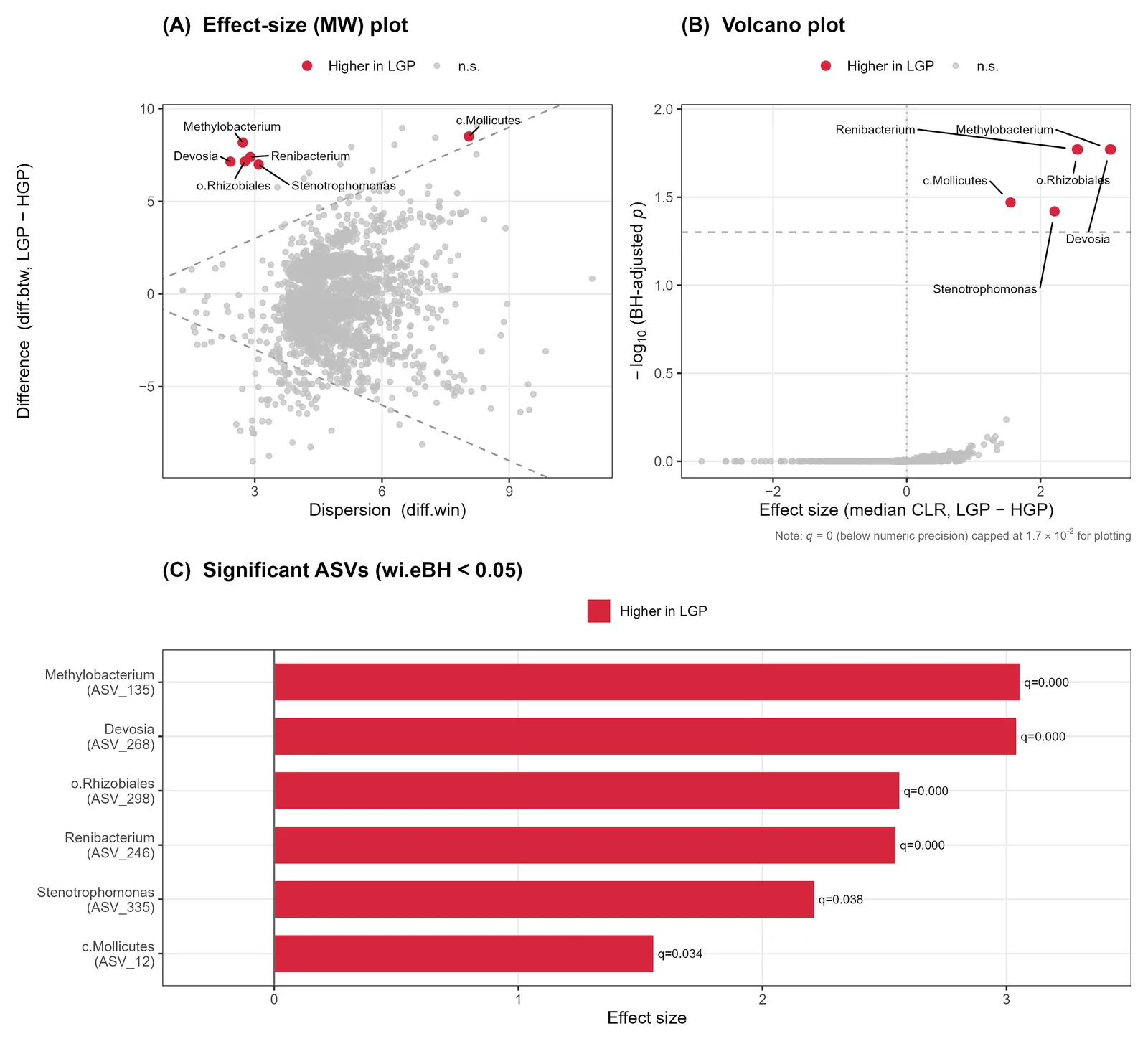
Compositional differential abundance analysis (ALDEx2) of gut microbiota between high- and low-growth-performance *P. leptodactylus*. Differential abundance of ASVs between the high-growth-performance (HGP, n = 5) and low-growth-performance (LGP, n = 5) groups, assessed with ALDEx2 (128 Monte-Carlo Dirichlet instances; centered log-ratio transformation with the all-feature denominator). (**A**) Effect-size (MW) plot of between-group difference (diff.btw, LGP − HGP) against within-group dispersion (diff.win); dashed diagonals mark |effect| = 1. (**B**) Volcano plot of ALDEx2 effect size against the Benjamini–Hochberg-adjusted *p* value from the expected Wilcoxon test (wi.eBH); the dashed horizontal line marks q = 0.05. (**C**) Effect sizes of the six ASVs that were significant at wi.eBH < 0.05, ordered by effect size and annotated with adjusted q values. Effect size was calculated based on median values of CLR-transformed ASV abundances obtained from ALDEx2 analysis. In all panels, colored points/bars denote significant ASVs colored by direction of enrichment; gray points are non-significant (n.s.). All six significant ASVs were enriched in the LGP group. Because four ASVs returned an adjusted *p* value below numerical precision (q ≈ 0), these values were capped at half of the smallest non-zero q for plotting purposes only (see caption note in panel (**B**)).

To cross-validate the findings with a compositionally aware approach, we re-analyzed the ASV table with ALDEx2 [21] (CLR transformation, all-feature denominator, 128 Monte-Carlo instances). Six ASVs were significantly differential between groups at a Benjamini–Hochberg-adjusted threshold of wi.eBH < 0.05, all of which were enriched in the low-growth-performance (LGP) group relative to the high-growth-performance (HGP) group (Figure 2A–C). These comprised ASVs assigned to *Methylobacterium* (ASV_135; effect = 3.05, q < 0.001), *Devosia* (ASV_268; effect = 3.03, q < 0.001), order Rhizobiales (ASV_298; effect = 2.60, q < 0.001), *Renibacterium* (ASV_246; effect = 2.55, q < 0.001), *Stenotrophomonas* (ASV_335; effect = 2.22, q = 0.038) and class Mollicutes (ASV_12; effect = 1.52, q = 0.034). In the HGP group, these ASVs were near-absent, whereas in the LGP group their CLR abundances were consistently elevated (Figure 2C). The effect size values displayed in Figure 2C were calculated using CLR normalized ASV abundances derived from ALDEx2 differential analysis. These differences represent statistical associations between community composition and growth phenotype and were not tested as causal effects.

#### 3.2.3. Analysis of Intestinal Microbiota Structure

**Figure 3:**
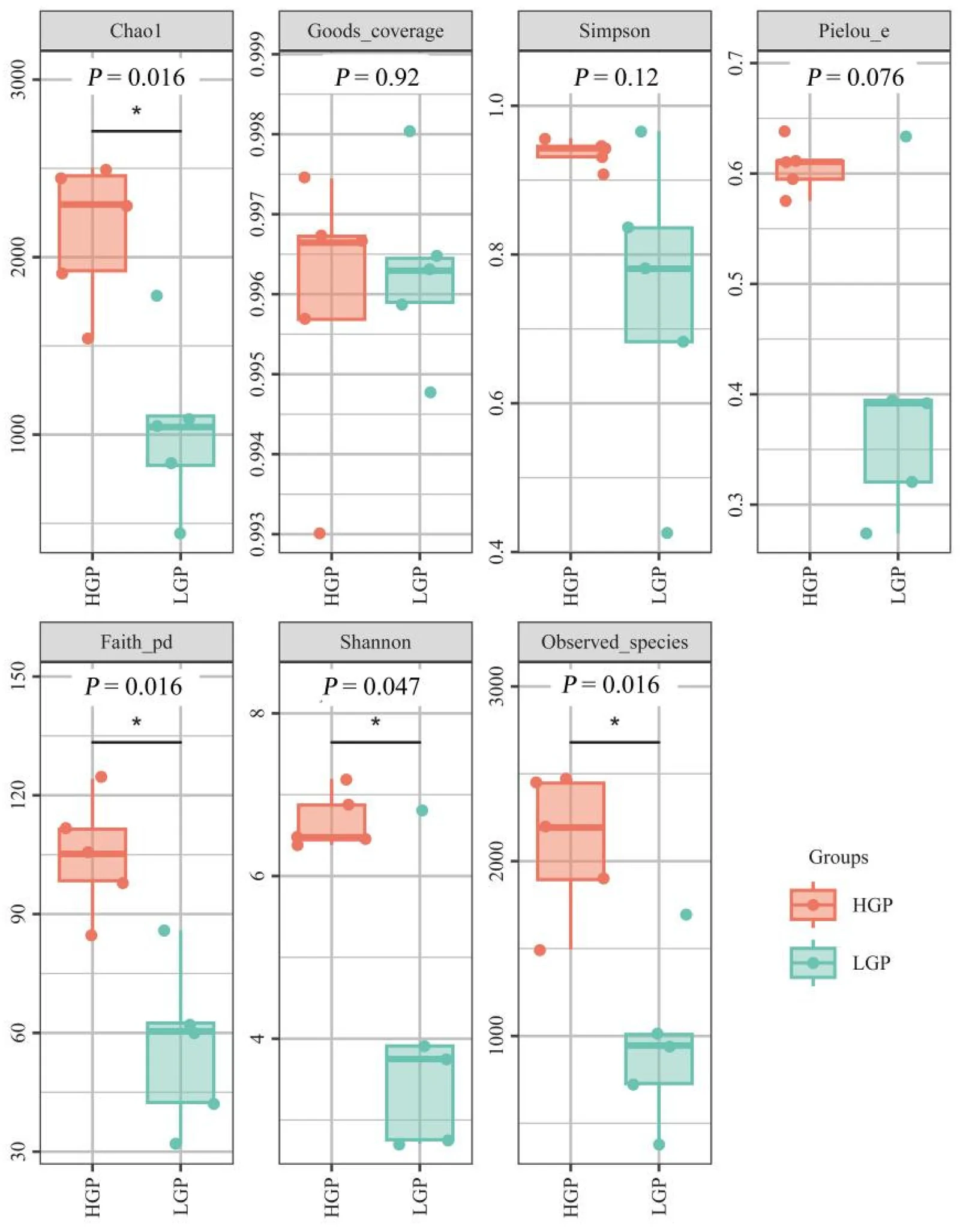
*α*-Diversity indices of HGP and LGP group. High-growth-performance group HGP (orange), low-growth-performance group LGP (green), *α*-diversity indices: Chao 1, Goods-coverage, Simpson, Pielou-e, Faith-pd, Shannon, and observed-species. The numbers under the diversity index label represent the *p* values of the Kruskal–Wallis test. When making pairwise group comparisons, the default is to display the significance markers from Dunn’s post hoc test. “* *p* < 0.05” indicates a significant difference.

**Figure 4:**
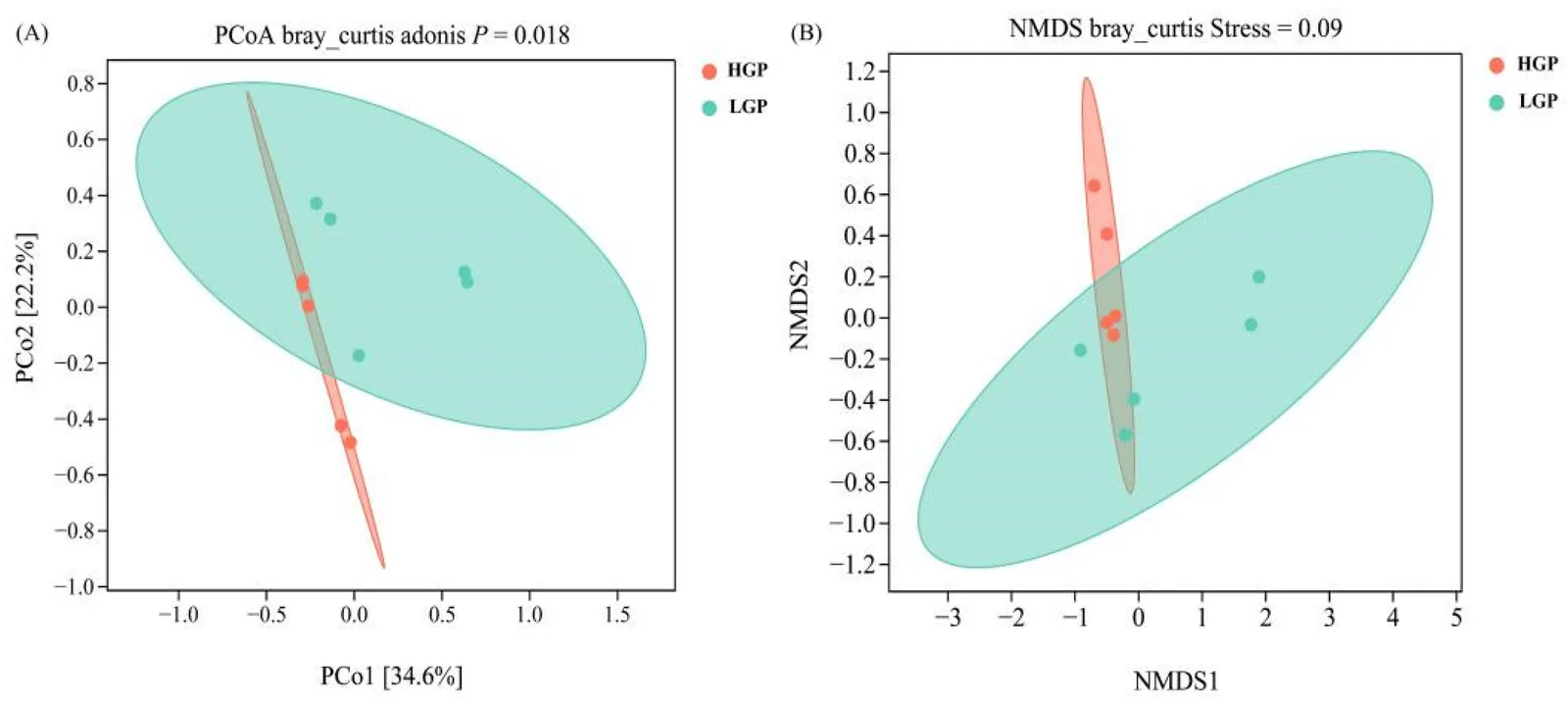
Intestinal microbiota structure analysis using PCoA and NMDS in HGP and LGP groups. (**A**) Principal coordinate analysis (PCoA) based on Bray–Curtis distance, adonis *p* = 0.018; (**B**) non-metric multidimensional scaling (NMDS) analysis based on Bray–Curtis distance. Ellipse denotes the 95% confidence interval of the overall ASV distribution in this plot.

Alpha diversity and beta diversity analyses were performed on the intestinal microbiota of *P. leptodactylus* in the HGP and LGP groups (Figure 3 and Figure 4). No significant difference was observed in the species coverage (Good_coverage) between the two groups (*p* = 0.92), with a value close to 1, and the rarefaction curve eventually levels off (Figure S1), indicating sufficient sequencing depth and high coverage. Significant differences were observed in the microbiota richness indices Chao1 and Observed species (*p* < 0.05), with more microbial species present in the intestines of male *P. leptodactylus* in the HGP group. The Shannon index, which reflects diversity, also showed a significant difference (*p* < 0.05), with the HGP group higher than the LGP group, while the Simpson index (*p* = 0.12) showed no significant difference. The evenness index Pielou_e (*p* = 0.076) showed no significant difference, indicating that the evenness of intestinal microbiota was comparable between the two groups. The HGP group exhibited higher intestinal microbiota diversity, and the phylogenetic diversity index Faith’s pd (*p* < 0.05) was significantly higher in the HGP group than in the LGP group, indicating that the microbial community in the HGP group had a broader distribution on the evolutionary tree and more diverse evolutionary lineages. Compared with the LGP group, the HGP group possessed more abundant microbial species, higher diversity, and broader evolutionary lineages, with greater community stability and functional evolutionary potential. These characteristics are conducive to jointly establishing a functionally rich, highly stable, and disturbance-resistant gut micro-ecosystem, potentially providing key microecological support for *P. leptodactylus* to efficiently utilize nutrients, alleviate physiological stress, and achieve rapid growth.

Beta diversity principal coordinate analysis (PCoA) of the intestinal microbiota from the two groups of *P. leptodactylus* (Figure 4A) showed that PCo1 contributed 34.6% and PCo2 contributed 22.2%. Compared with the HGP group, the intestinal microbiota structure of the LGP group showed significant changes. PERMANOVA indicated a significant difference in community structure between the HGP and LGP groups (Adonis: pseudo-F = 2.492, R^2^ = 0.237, *p* = 0.018, 999 permutations, Bray–Curtis distance); the betadisper test showed no significant difference in dispersion between groups (*p* = 0.256), suggesting that the observed difference is mainly due to a shift in the centroids of community composition rather than differences in within-group dispersion. NMDS analysis results (Figure 4B) confirmed significant differences between groups, with a stress value of 0.009, which was less than 0.2, indicating excellent model fit and reliable results.

#### 3.2.4. Differences in Intestinal Microbiota Composition

**Figure 5:**
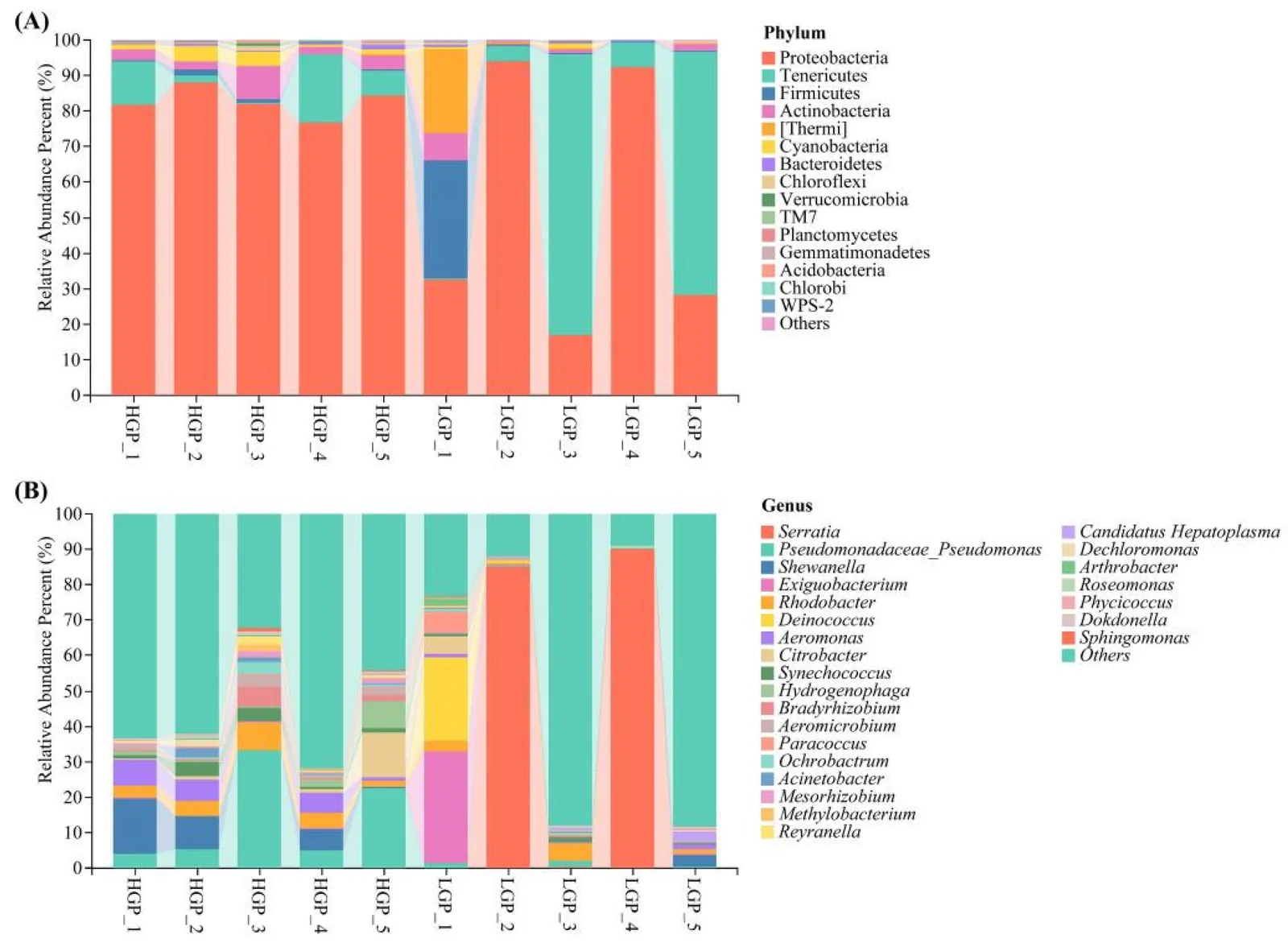
Composition and relative abundance of intestinal microbiota at the phylum and genus levels in HGP and LGP groups. (**A**) Top 15 phyla by relative abundance; (**B**) top 25 genera by relative abundance.

At the phylum level (Figure 5A), the top five phyla in terms of relative abundance were Proteobacteria, Tenericutes, Firmicutes, Actinobacteria, and Thermi. The top 15 phyla in the HGP and LGP groups accounted for more than 99.79% of the total annotated species abundance. At the genus level (Figure 5B), the top 10 genera in terms of relative abundance were *Serratia*, *Pseudomonas*, *Shewanella*, *Exiguobacterium*, *Rhodobacter*, *Deinococcus*, *Aeromonas*, *Citrobacter*, *Synechococcus*, and *Hydrogenophaga*. The top 25 genera accounted for more than 50.61% of the total annotated species abundance, indicating that these genera represent the core components of the intestinal microbiota.

**Figure 6:**
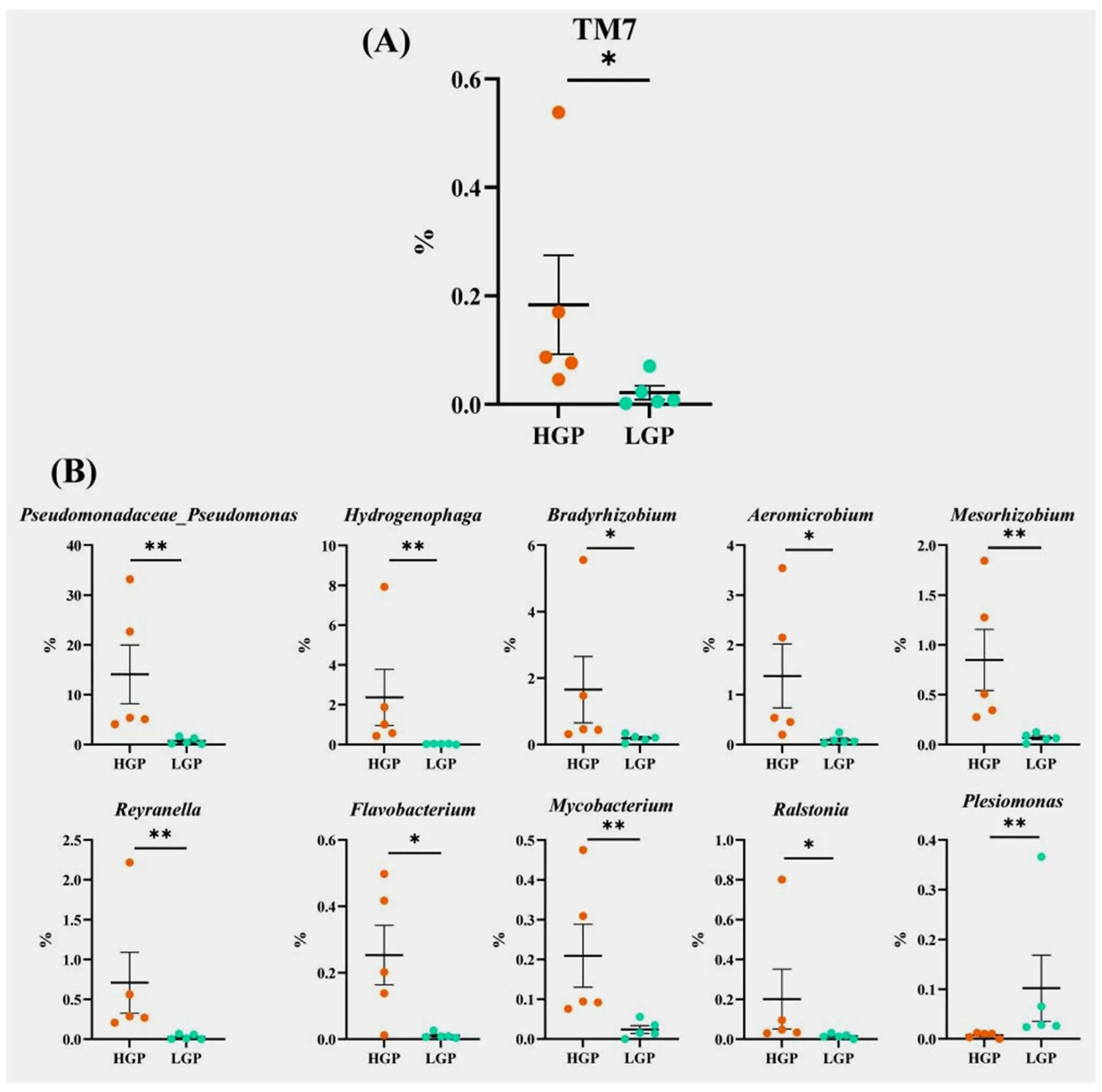
Significant differences in intestinal microbiota at the phylum (**A**) and genus (**B**) levels between HGP and LGP groups. *p* < 0.05 (*), *p* < 0.01 (**) (BH-corrected; Mann–Whitney U test).

The differentially abundant phylum of intestinal microbiota was TM7 (Figure 6A), with higher abundance in the HGP group than in the LGP group. A total of 10 genera exhibited significant differences or differential trends between the two groups (Figure 6B). Compared with the LGP group, the relative abundances of *Pseudomonas*, *Hydrogenophaga*, *Mesorhizobium*, *Reyranella*, *Mycobacterium*, and other genera in the HGP group were significantly increased (*p* < 0.01), the relative abundances of *Flavobacterium*, *Ralstonia*, *Bradyrhizobium*, and *Aeromicrobium* were significantly increased (*p* < 0.05), while the relative abundance of *Plesiomonas* was significantly decreased (*p* < 0.01).

### 3.3. Analysis of Differences in Intestinal Microbiota Metabolic Pathways

**Figure 7:**
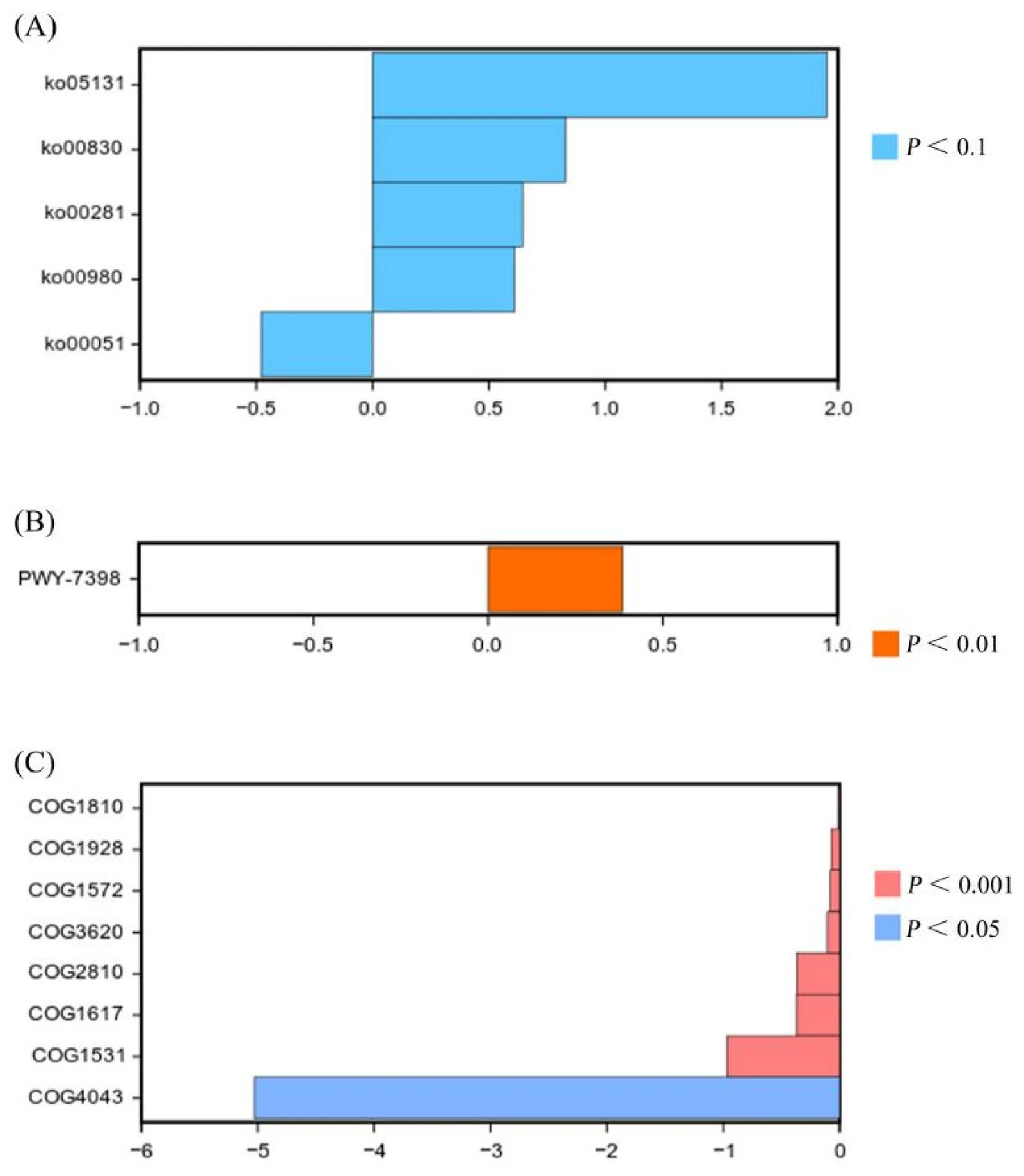
Analysis of differences in metabolic pathways. (**A**) KEGG pathway analysis, HGP group as the upregulated group, LGP group as the control group, *p* < 0.1 indicates a trend toward difference; (**B**) MetaCyc pathway analysis, HGP group as the upregulated group, LGP group as the control group, *p* < 0.01 indicates a significant difference; (**C**) COG functional gene analysis, HGP group as the upregulated group, LGP group as the control group, *p* < 0.05 indicates a significant difference, *p* < 0.001 indicates a highly significant difference. These *p*-values are all FDR-adjusted *p*-values.

Compared to the LGP, the HGP in the following intestinal microbiota-mediated pathways: Shigellosis (ko05131), Retinol metabolism (ko00830), Geraniol degradation (ko00281), and Metabolism of xenobiotics by cytochrome P450 (ko00980) showed an upward trend (log2FC > 0, *p* < 0.10), while the fructose and mannose metabolism pathway (ko00051) showed a downregulated trend (log2FC < 0, *p* < 0.10) (Figure 7A).

Compared to the LGP group, the HGP group exhibited significantly upregulated microbiota-mediated coumarin biosynthesis pathways (PWY-7398) (log2FC = 0.39, *p* < 0.01) (Figure 7B). Its actual connection needs to be confirmed by further experimental studies.

At the functional gene level, compared to the LGP group, the abundance of eight functional genes in the intestinal microbiota of the HGP group decreased (Figure 7C). These eight functional genes were: uncharacterized conserved proteins/selenoproteins, DUF166 family (COG1810); dolichyl-phosphate-mannose--protein O-mannosyl transferase (COG1928); uncharacterized protein, contains cell adhesion-related (CARDB) domains (COG1572); predicted transcriptional regulator, contains an XRE-type HTH domain (archaeal members contain CBS pair) (COG3620); predicted type IV restriction endonuclease (COG2810); threonylcarbamoyladenosine modification (KEOPS) complex, Cgi121 subunit (COG1617); uncharacterized conserved protein, UPF0248 family (COG1531); ASC-1 homology (ASCH) domain, predicted RNA-binding domain (COG4043).

## 4. Discussion

In this study, an extreme-phenotype sampling strategy was employed to compare the intestinal microbiota of male *P. leptodactylus* exhibiting divergent growth performance. Under identical rearing conditions, individuals in the high-growth-performance (HGP) group displayed substantially greater weight gain (11.84–18.50 g) and length increase (0.40–1.60 cm) than the low-growth-performance (LGP) group (weight gain: 0.76–1.25 g; length increase: 0.10–0.40 cm), and significantly higher weight gain rate and specific growth rate (*p* < 0.01), confirming marked inter-individual variation. Because the groups were deliberately chosen from the tails of the growth distribution, these differences are expected and do not constitute an independent discovery; subsequent intestinal microbiota analyses were built on this extreme-phenotype framework. All microbiota–phenotype relationships reported herein should be interpreted as statistical associations, as the cross-sectional design cannot establish causality.

Rarefaction analysis and Good’s coverage values (close to 1, *p* = 0.92 between groups) indicated sufficient sequencing depth. Venn analysis revealed that the HGP group harbored far more unique ASVs (7840; 65.97%) than the LGP group (3383; 28.46%). Alpha-diversity indices showed significantly higher Chao1, observed species, Shannon and Faith’s PD in the HGP group (*p* < 0.05), while Pielou’s evenness was comparable. Higher diversity has often been linked to functional redundancy and resilience against disturbances [22], characteristics that may favor efficient nutrient utilization and lower physiological stress, consistent with trends reported for Pacific white shrimp [23].

It is noteworthy that the association between crustacean microbiome diversity and growth performance has been inconsistent in previous studies. In this study, higher α-diversity was observed in the HGP group, which is consistent with several studies reporting that “high-growth individuals are accompanied by richer gut microbiome diversity.” However, recent studies also suggest that differences in growth performance are more associated with differences in the abundance of specific genera rather than overall *α*-diversity—for example, Uengwetwanit et al. (2020) [24] found in their study on the tiger shrimp that growth differences between high-weight and low-weight individuals were mainly explained by the abundance differences in specific genera such as Brevibacillus and Fusibacter, rather than systematic changes in overall *α*-diversity. Moreover, Landsman et al. (2019) [25] in their study on the Pacific white shrimp indicate that under certain conditions, changing the gut microbiome composition does not necessarily translate into statistically significant growth performance differences, suggesting that the association between the two may be influenced by species, developmental stage, aquaculture environment, and analytical method differences. Therefore, the observed *α*-diversity differences between the HGP and LGP groups in this study should be interpreted cautiously and should not be extrapolated as a causal rule that “microbial diversity drives growth,” but rather regarded as an exploratory finding that requires further validation.

Beta-diversity analyses (PCoA, NMDS, PERMANOVA *p* = 0.018) confirmed a genuine shift in community structure between groups, with comparable within-group dispersion.

### 4.1. Core Indicator ASVs Identified by ALDEx2

Using the compositional data analysis tool ALDEx2, we identified six ASVs that were significantly enriched in the LGP group and nearly absent in the HGP group. These ASVs were assigned to *Methylobacterium*, *Devosia*, unclassified Rhizobiales, *Renibacterium*, *Stenotrophomonas* and Mollicutes, forming a microbial fingerprint robustly associated with poor growth performance. Their roles should be interpreted from multiple perspectives.

*Methylobacterium* and partial members of *Rhizobiales* are well-characterized facultative methylotrophs that scavenge methanol, methylamine and other one-carbon molecules for cellular anabolism, whereas *Devosia* mainly acts as heterotrophic bacteria with sporadic weak methylotrophic capacity. Methylotrophic metabolic pathways consume higher cellular energy input, leading to intrinsically slower growth rates compared with common copiotrophic heterobacteria [26]. Accordingly, methylotrophic populations are readily outcompeted by fast-growing heterotrophs under nutrient-rich conditions with abundant multi-carbon substrates. The significant enrichment of these methylotrophy-related taxa in LGP crayfish gut indicates an imbalanced intestinal nutritional microenvironment in low-growth individuals, which may further interfere with nutrient digestion and absorption efficiency of *P. leptodactylus.* Their enrichment in the LGP group may therefore reflect an altered gut environment—perhaps with reduced availability of easily fermentable carbohydrates or changes in intestinal transit—that allows these specialists to persist, rather than indicating a direct growth-suppressive effect. *Renibacterium* is a known causative agent of bacterial kidney disease in salmonids, yet subclinical infections with carrier fish remaining asymptomatic are well documented [27]. Its accumulation in poorly growing crayfish may be analogous to such a carrier state, but the pathogenic significance cannot be determined from amplicon data alone and would require absolute quantification together with assessment of host immune status. *Stenotrophomonas* commonly blooms under dysbiotic conditions [28], and its dominance in the LGP group likely signals intestinal dysbiosis. Members of Mollicutes have been shown to participate in cellulose degradation and nitrogen recycling in some aquatic herbivores [29], yet their functional roles in crayfish gut remain unresolved. Collectively, these six ASVs may act partially as indicators of an unfavorable gut environment and partially through metabolic interactions or opportunistic pathogenesis; however, the causal direction awaits validation in time-series studies.

### 4.2. Supplementary Evidence from Phylum- and Genus-Level Shifts

At higher taxonomic levels, the overall compositional shift corroborates the ALDEx2 findings. The phylum TM7 (Saccharibacteria) was significantly more abundant in the HGP group. TM7 can suppress potential pathogens through an episymbiotic lifestyle [30] and has been shown to attenuate inflammatory bone loss in a mouse periodontitis model [31] and to modulate host fatty acid metabolism in insects [32]. Its enrichment may thus contribute to intestinal homeostasis via immunomodulation and metabolite-mediated pathways.

At the genus level, ten genera were significantly enriched in the HGP group, forming a potential functional network. *Pseudomonas* and *Hydrogenophaga* can degrade organic pollutants and oxyanions, thereby detoxifying the microenvironment [33,34,35]. *Mesorhizobium* and *Bradyrhizobium* can mobilize inert nitrogen and potassium, enhancing host nutrient acquisition [36,37]. *Reyranella* has been implicated in carbon and nitrogen fixation [38], quorum quenching of pathogens [39], and growth promotion in tilapia [40]. *Flavobacterium*, *Aeromicrobium* and *Ralstonia* synergistically decompose macromolecular organic matter such as polysaccharides and proteins, facilitating nutrient cycling and energy harvest [41,42,43,44]; among them, *Flavobacterium* can degrade algal polysaccharides and produce short-chain fatty acids that contribute to gut energy homeostasis and inflammation suppression [42]. Conversely, *Plesiomonas* was significantly reduced in the HGP group. This genus includes known enteric pathogens [45,46], and its reduction may lower infection risk and immune energy expenditure. Although genus-level functional inference is limited by 16S rRNA gene resolution, the overall picture is one of a strengthened beneficial network and suppressed potential pathogens in high-growth individuals.

### 4.3. Functional Prediction as Extended Perspective and Its Caveats

PICRUSt2-based functional prediction offered an extended view beyond taxonomic composition but carries the highest uncertainty [20]. At the KEGG pathway level, retinol metabolism (ko00830) showed an upward trend in the HGP group. This pathway is linked to vitamin A synthesis and promotes intestinal barrier integrity and immune homeostasis [47], as also reported in the intestinal microbiota of the Chinese pond turtle [48]. The upward trend of the shigellosis pathway (ko05131) may reflect a state of immune alertness; in southern catfish, activation of this microbiota-mediated pathway enhances host inflammatory defense and pathogen clearance in response to *Plesiomonas shigelloides* infection [49]. Considering the enrichment of *Plesiomonas* and *Renibacterium* in the LGP group, the mild upregulation of this pathway in the HGP group might indicate a primed immune status. Upregulation of geraniol degradation and cytochrome P450-mediated xenobiotic metabolism suggests heightened detoxification potential in the HGP intestinal microbiota [50,51]. In red swamp crayfish (*Procambarus clarkii*), the intestinal microbial P450 pathway has been demonstrated to alleviate tissue oxidative damage caused by mercury [52].

In the MetaCyc database, the coumarin biosynthesis pathway (PWY-7398) was significantly upregulated in the HGP group (*p* < 0.01). A coumarin derivative, N2905, has been shown to reduce white spot syndrome virus (WSSV) copy number in shrimp larvae by over 90% and improve post-infection survival [53]. The upregulation of this pathway hints that the HGP intestinal microbiota may possess enhanced potential for synthesizing antiviral compounds.

At the COG functional gene level, eight gene features were downregulated in the HGP group, spanning host–microbe interaction, energy metabolism, and gene expression regulation. COG1810 (DUF166 family) may be involved in carbon monoxide dehydrogenase maturation [54]; COG1572 contains a CARDB domain potentially mediating bacterial-host adhesion [55]; COG4043 encodes a predicted RNA-binding domain [56]; COG1928 participates in post-translational modification and calcium signaling [57]; COG3620 contains a CBS domain related to GTP synthesis [58]; and COG1617 is part of the KEOPS complex ensuring translational accuracy [59]. The downregulation of these genes may collectively reflect a more “economical” microbial metabolism, reducing energy expenditure on adhesion, defense and unnecessary transcriptional regulation, thereby channeling more substrates toward host growth. Nonetheless, these predictions represent genomic potential rather than realized metabolic activity, and the annotation accuracy for non-model aquatic invertebrates remains limited [20]. These functional interpretations must be regarded as hypotheses requiring multi-omics validation.

### 4.4. Limitations

This study has several significant limitations: First, the cross-sectional extreme phenotype design, which classifies high- and low-growth groups solely based on body weight, does not allow causal inference, and the observed microbial differences can only be regarded as statistically associated with growth traits. Second, the small sample size of only five individuals per group may limit statistical power and the accuracy of effect size estimates. Third, only male *P. leptodactylus* were collected; although this excludes sex confounding and males constitute the main production population, it restricts the generalization of the results to females and reproductive scenarios. Fourth, although rearing near the natural habitat reduced large-scale geographic variation, the positions of the net cages within the ponds were not completely randomized; while each *P. leptodactylus* was reared individually to avoid pseudoreplication, microenvironmental differences such as water flow, shading, and substrate could still introduce residual confounding. Additionally, sampling, experiments, and data analysis were not blinded, which may result in observer bias. Fifth, the 21-day experimental period was relatively short and insufficient to distinguish stable core microbiota from transient community fluctuations. Moreover, PICRUSt2 functional predictions only reflect the genomic potential of the microbiota and are susceptible to annotation bias in non-model hosts; actual metabolic activities require validation with metatranscriptomics and metabolomics. In summary, the current results should be regarded as exploratory and hypothesis-generating rather than definitive conclusions; future studies using microbiome manipulation and longitudinal sampling are needed to elucidate the causal relationship between the microbiome and host growth performance.

## 5. Conclusions

By comparing intestinal microbiota from the growth extremes of *P. leptodactylus*, ALDEx2 analysis identified a core set of ASVs (methylotrophs, *Renibacterium*, *Stenotrophomonas* and Mollicutes) robustly associated with low growth performance, complemented by phylum- and genus-level enrichment of TM7 and beneficial bacterial networks and reduction in *Plesiomonas*. Functional prediction further suggests differential potentials in antimicrobial defense, detoxification and immune pathways. These associations provide candidate microbial targets for probiotic screening and aquaculture management, while causal verification remains the critical next step.

## Figures and Tables

**Table 1:** Grouping, samples, initial weight, final weight, and weight difference.

Group	Sample	Initial Weight (g)	Final Weight (g)	Weight Difference Value (g)
HGP	A1	51.00	69.50	18.50
	A2	47.50	63.50	16.00
	A3	47.00	62.30	15.30
	A4	45.50	57.50	12.00
	A5	49.50	61.34	11.84
LGP	B1	42.09	42.86	0.77
	B2	40.59	41.35	0.76
	B3	45.00	45.81	0.81
	B4	47.50	48.55	1.05
	B5	44.00	45.25	1.25

Note: HGP = high-growth-performance group; LGP = low-growth-performance group. Initial weight (g): body weight of crayfish at the start of the experiment; final weight (g): body weight of crayfish at the end of the experiment. Weight difference value (g): the difference between final weight and initial weight of crayfish in each treatment.

**Table 2:** Grouping, samples, initial length, final length, and length difference.

Group	Sample	Initial Length (cm)	Final Length (cm)	Length Difference Value (cm)
HGP	A1	11.20	12.80	1.60
	A2	11.30	12.00	0.70
	A3	11.40	12.30	0.90
	A4	11.40	12.30	0.90
	A5	12.00	12.40	0.40
LGP	B1	10.90	11.20	0.30
	B2	10.80	11.10	0.30
	B3	11.80	11.90	0.10
	B4	11.80	12.20	0.40
	B5	11.10	11.40	0.30

Note: HGP = high-growth-performance group; LGP = low-growth-performance group. Initial length (cm): body length of crayfish at the start of the experiment; final length (cm): body length of crayfish at the end of the experiment. Length difference value (cm): the difference between final length and initial length of crayfish in each treatment.

**Table 3:** Body length growth rate (BLGR, %), weight gain rate (WGR, %), specific growth rate (SGR, %/d).

Group	Weight Difference Value (g)	Length Difference Value (cm)	Weight Gain Rate (WGR, %)	Body Length Growth Rate (BLGR, %)	Specific Growth Rate (SGR, %/d)
HGP	14.73 ± 2.83	0.90 ± 0.44	30.55 ± 5.19	7.92 ± 4.02	1.26 ± 0.19
LGP	0.93 ± 0.22	0.28 ± 0.11	2.11 ± 0.44	2.49 ± 0.96	0.10 ± 0.02

Note: HGP = high-growth-performance group; LGP = low-growth-performance group. Weight difference value (g) refers to the net weight gain during the experiment; length difference value (cm) refers to the net body length increase during the experiment; weight gain rate (WGR, %) is the percentage of weight gain relative to initial weight; body length growth rate (BLGR, %) is the percentage of body length increase relative to initial length; specific growth rate (SGR, %/d) is the daily growth rate of body weight. Values are presented as mean ± SD.

## Data Availability

The raw 16S rRNA gene sequencing data (FASTQ files) and sample metadata generated in this study have been deposited in the NCBI Sequence Read Archive (SRA) under BioProject accession number PRJNA1495575 (https://www.ncbi.nlm.nih.gov/bioproject/PRJNA1495575, accessed on 15 July 2026), with a scheduled public release date of 30 September 2026. The ASV table, representative sequences, taxonomy table, sample metadata, QIIME2 analysis commands, and statistical scripts are available from the corresponding author upon reasonable request.

## Supplementary Materials

The following supporting information can be downloaded at: https://www.mdpi.com/article/10.3390/microorganisms14081739/s1, Figure S1: Shannon index rarefaction curves of intestinal microbiota in high growth performance (HGP) and low growth performance (LGP) groups of Pontastacus leptodactylus along gradient sequencing depth; Figure S2: Boxplot of significantly differential genera screened by ALDEx2 analysis; Figure S3: LEfSe cladogram and LDA score analysis of differential bacterial taxa; File S1: ARRIVE 2.0 checklist for animal experiment design and welfare assessment of *Pontastacus leptodactylus*.
