# Supplementary material for: Effects of Intestinal Microbiota on Individual Growth and Development of Pontastacus leptodactylus
Source: Microorganisms. 2026 Aug 7;14(8):1739. doi: 10.3390/microorganisms14081739 (PMC13515472; doi:10.3390/microorganisms14081739)
Supplement: Supplementary file 1 [file microorganisms-14-01739-s001.zip › File S1.pdf]

NOTE: Please save this file locally before filling in the table, DO NOT work on the file within your internet browser as changes will not be saved. Adobe Acrobat Reader (available free here) is recommended for completion.

The ARRIVE guidelines 2.0: author checklist

The ARRIVE Essential 10

These items are the basic minimum to include in a manuscript. Without this information, readers and reviewers cannot assess the reliability of the findings.

Item		Recommendation	Section/line number, or reason for not reporting
**Study design**	1	For each experiment, provide brief details of study design including:<br>a. The groups being compared, including control groups. If no control group has been used, the rationale should be stated.<br>b. The experimental unit (e.g. a single animal, litter, or cage of animals).	, n = 5) and Low Growth Performance (LGP, n = 5) male Pontastacus leptodactylus, selected by extreme-phenotype sampling from the same pond cohort. No separate control group; LGP serves as the comparator. Study is explicitly repositioned as an exploratory extreme-phenotype comparison.Section 2.1, 2.3<br>iduals was housed in a separate hanging cage (HGP: cages A1–A5; LGP: cages B1–B5); no two sequenced animals shared a cage.Section 2.1;<br>Supplementary Table S1
**Sample size**	2	a. Specify the exact number of experimental units allocated to each group, and the total number in each experiment. Also indicate the total number of animals used.<br>b. Explain how the sample size was decided. Provide details of any *a priori* sample size calculation, if done.	across 23 complete cages met inclusion criteria. From these, 10 individuals were selected for 16S rRNA sequencing: HGP n = 5, LGP n = 5. Total animals used in the experiment: 75.Section 2.1;<br>Table 1–3<br>RNA studies in crustaceans. The extreme-phenotype design was chosen to maximise detection sensitivity given the small n. This limitation is acknowledged in Section 4.5.Section 2.1;<br>Section 4.5
**Inclusion and exclusion criteria**	3	a. Describe any criteria used for including and excluding animals (or experimental units) during the experiment, and data points during the analysis. Specify if these criteria were established *a priori*. If no criteria were set, state this explicitly.<br>b. For each experimental group, report any animals, experimental units or data points not included in the analysis and explain why. If there were no exclusions, state so.<br>c. For each analysis, report the exact value of *n* in each experimental group.	uded), escape due to cage flooding (n = 2 excluded), death during rearing (n = 1). For sequencing, individuals were selected from the top and bottom 5 of the weight-gain distribution from qualifying cages.Section 2.1<br>d from bioinformatic analysis due to insufficient read depth after quality filtering. Final analysis: n = 5 HGP, n = 5 LGP.Section 2.1;<br>Section 2.5;<br>Supplementary Table S1<br>Growth performance (Table 1–3): HGP n = 5, LGP n = 5. Alpha/beta diversity and differential abundance (Figures 2–7): HGP n = 5, LGP n = 5. All analyses use the same 10 individuals. Tables 1–3;<br>Figures 2–7
**Randomisation**	4	a. State whether randomisation was used to allocate experimental units to control and treatment groups. If done, provide the method used to generate the randomisation sequence.<br>b. Describe the strategy used to minimise potential confounders such as the order of treatments and measurements, or animal/cage location. If confounders were not controlled, state this explicitly.	[illegible]ion sequence.<br>Randomisation was NOT used. Animals were placed into cages in the order of collection from the pond. Groups (HGP/LGP) were defined post hoc based on weight-gain ranking after the 21-day rearing period. This is explicitly stated in the revised manuscript. Section 2.1<br>omised. All cages were suspended in the same pond under identical management conditions (same feed, water source, photoperiod). The potential for spatial confounding by cage position is acknowledged as a limitation in Section 4.5. Section 2.1;<br>Section 4.5
**Blinding**	5	Describe who was aware of the group allocation at the different stages of the experiment (during the allocation, the conduct of the experiment, the outcome assessment, and the data analysis).	all investigators during sample collection, laboratory processing, and data analysis, as groups were defined by the weight-gain ranking used to select animals. This is explicitly stated in the revised manuscript. The associated risk of observer bias is acknowledged in Section 4.5. Section 2.1;<br>Section 4.5
**Outcome measures**	6	a. Clearly define all outcome measures assessed (e.g. cell death, molecular markers, or behavioural changes).<br>b. For hypothesis-testing studies, specify the primary outcome measure, i.e. the outcome measure that was used to determine the sample size.	ys (PICRUSt2: KEGG, MetaCyc, COG). Growth metrics (WGR, SGR, BLGR) are descriptive only (selection criterion; not used for inferential testing). Section 2.3, 2.5, 2.6;<br>Figures 3–7<br>ys (PICRUSt2: KEGG, MetaCyc, COG). Growth metrics (WGR, SGR, BLGR) are descriptive only (selection criterion; not used for inferential testing). Section 2.3, 2.5, 2.6;<br>Figures 3–7
**Statistical methods**	7	a. Provide details of the statistical methods used for each analysis, including software used.<br>b. Describe any methods used to assess whether the data met the assumptions of the statistical approach, and what was done if the assumptions were not met.	6); R vegan package. Differential abundance: ALDEx2 (CLR, Welch t-test, BH-FDR) + ANCOM-II ($W \geq 0.7$); R. Functional pathways: ALDEx2 on PICRUSt2 output; BH-FDR correction. Section 2.6;<br>Section 3.2.3<br>ed. For compositional data (microbiota), ALDEx2 (CLR transformation) was used to address compositionality without normality assumptions. Multivariate dispersion was assessed by betadisper before interpreting PERMANOVA. Section 2.6
**Experimental animals**	8	a. Provide species-appropriate details of the animals used, including species, strain and substrain, sex, age or developmental stage, and, if relevant, weight.<br>b. Provide further relevant information on the provenance of animals, health/immune status, genetic modification status, genotype, and any previous procedures.	ed from commercial aquaculture stock). Initial body weight: HGP 28.04 ± 3.27 g, LGP 27.76 ± 3.01 g (mean ± SD). Initial body length: HGP 10.38 ± 0.55 cm, LGP 10.30 ± 0.52 cm. Section 2.1;<br>Tables 1–2<br>althy at stocking (no visible lesions, normal appendages). Genetic modification status: none. No previous experimental procedures. Animals underwent a 7-day acclimation period before the experiment. Section 2.1
**Experimental procedures**	9	For each experimental group, including controls, describe the procedures in enough detail to allow others to replicate them, including:<br>a. What was done, how it was done and what was used.<br>b. When and how often.<br>c. Where (including detail of any acclimatisation periods).<br>d. Why (provide rationale for procedures).	by rapid destruction of suboesophageal ganglion; death confirmed by absence of reflex. Intestinal contents collected immediately; stored at ?80 °C. Section 2.1, 2.2
**Results**	10	For each experiment conducted, including independent replications, report:<br>a. Summary/descriptive statistics for each experimental group, with a measure of variability where applicable (e.g. mean and SD, or median and range).<br>b. If applicable, the effect size with a confidence interval.	, SGR 2.07 ± 0.32%/d, LGP weight gain 1.01 ± 0.18 g, WGR 3.65 ± 0.65%, SGR 0.17 ± 0.03%/d. Alpha diversity (mean ± SD per group) reported in Figure 3. All values presented as mean ± SD throughout. Tables 1–3;<br>Figure 3<br>.55 (95% CI [0.59, 17.7]), q < 0.001, Methylobacterium effect = 3.05 (95% CI [0.56, 23.8]), q < 0.001. Full effect sizes and 95% CIs for all taxa in Supplementary Table S2. Section 3.2.3;<br>Section 3.2.2;<br>Supplementary Table S2

The Recommended Set

These items complement the Essential 10 and add important context to the study. Reporting the items in both sets represents best practice.

Item		Recommendation	Section/line number, or reason for not reporting
**Abstract**	11	Provide an accurate summary of the research objectives, animal species, strain and sex, key methods, principal findings, and study conclusions.	[illegible]associations requiring further validation.
**Background**	12	a. Include sufficient scientific background to understand the rationale and context for the study, and explain the experimental approach.	[illegible] ptodactylus as an emerging aquaculture species in Xinjiang, the role of gut microbiota in crustacean growth, and the knowledge gap for this species. Condensed by ~25% in revision to remove redundant paragraphs. Section 1
		b. Explain how the animal species and model used address the scientific objectives and, where appropriate, the relevance to human biology.	[illegible] ptodactylus as an emerging aquaculture species in Xinjiang, the role of gut microbiota in crustacean growth, and the knowledge gap for this species. Condensed by ~25% in revision to remove redundant paragraphs. Section 1
**Objectives**	13	Clearly describe the research question, research objectives and, where appropriate, specific hypotheses being tested.	[illegible]esis pre-registered. Section 1;Section 2.1
**Ethical statement**	14	Provide the name of the ethical review committee or equivalent that has approved the use of animals in this study, and any relevant licence or protocol numbers (if applicable). If ethical approval was not sought or granted, provide a justification.	Provide the name of the ethical review committee or equivalent that has approved the use of animals in this study, and any relevant licence or protocol numbers.<br>Ethical approval provided by the Animal Welfare and Ethics Committee of Xinjiang Agricultural University. Protocol number: 2024057. Section 2.1;<br>Institutional Review Board Statement
**Housing and husbandry**	15	Provide details of housing and husbandry conditions, including any environmental enrichment.	[illegible]ation before experiment start. Section 2.1
**Animal care and monitoring**	16	a. Describe any interventions or steps taken in the experimental protocols to reduce pain, suffering and distress.	[illegible] Euthanasia was performed by rapid destruction of the suboesophageal ganglion, consistent with recommended crustacean humane killing methods. Chilled anhydrous transport (~8 h) was used to suppress metabolism and reduce stress. Section 2.2
		b. Report any expected or unexpected adverse events.	[illegible]<br>Unexpected adverse events: 2 individuals escaped when heavy summer rains flooded one cage's feeding port; 1 individual died following compression injury sustained at stocking. These are reported in the animal accounting (Section 2.1). Section 2.1
		c. Describe the humane endpoints established for the study, the signs that were monitored and the frequency of monitoring. If the study did not have humane endpoints, state this.	[illegible] on, visible severe injury. Monitoring: daily visual inspection of all cages for health status, moulting, and mortality. Animals meeting humane endpoint criteria were euthanised immediately. Section 2.1, 2.2
**Interpretation/ scientific implications**	17	a. Interpret the results, taking into account the study objectives and hypotheses, current theory and other relevant studies in the literature.	[illegible]hout. Discussion contextualises findings against published crustacean microbiome literature, including studies showing inconsistent microbiota-growth associations (Uengwetwanit et al. 2020; Landsman et al. 2019). Section 4
		b. Comment on the study limitations including potential sources of bias, limitations of the animal model, and imprecision associated with the results.	[illegible]ype selection bias; (3) no randomisation or blinding; (4) no extraction-blank controls; (5) cage positions not randomised; (6) 21-day trial may not capture stable microbiota. Cross-sectional design precludes causal inference. Section 4.5
**Generalisability/ translation**	18	Comment on whether, and how, the findings of this study are likely to generalise to other species or experimental conditions, including any relevance to human biology (where appropriate).	[illegible] o other species or experimental conditions.<br>Findings are specific to male P. leptodactylus under the described aquaculture conditions in Altay, Xinjiang. Generalisation to other crustacean species, sexes, developmental stages, or environments requires further study. No human biology relevance is claimed.
**Protocol registration**	19	Provide a statement indicating whether a protocol (including the research question, key design features, and analysis plan) was prepared before the study, and if and where this protocol was registered.	Provide a statement indicating whether a protocol was prepared before the study, and if and where this protocol was registered.<br>No pre-study protocol was registered. This is an exploratory observational study; the absence of pre-registration is acknowledged as a limitation consistent with the exploratory design. Section 4.5
**Data access**	20	Provide a statement describing if and where study data are available.	[illegible]ntary File S2. Data Availability Statement
**Declaration of interests**	21	a. Declare any potential conflicts of interest, including financial and non-financial. If none exist, this should be stated.	Declare any potential conflicts of interest, including financial and non-financial.<br>The authors declare no conflict of interest. Conflicts of Interest
		b. List all funding sources (including grant identifier) and the role of the funder(s) in the design, analysis and reporting of the study.	[illegible]<br>This research was funded by the Science and Technology Plan Project of Altay Prefecture, Xinjiang (Grant number: 202405). The funder had no role in study design, data collection, analysis, interpretation, or manuscript preparation.
